# The AabHLH35 Transcription Factor Identified from *Anthurium andraeanum* is Involved in Cold and Drought Tolerance

**DOI:** 10.3390/plants8070216

**Published:** 2019-07-11

**Authors:** Li Jiang, Xingkai Tian, Shuting Li, Yanxia Fu, Jiaojun Xu, Guangdong Wang

**Affiliations:** Department of Horticulture, Nanjing Agricultural University, Nanjing 210095, China

**Keywords:** *Anthurium andraeanum* Lind., leaf color mutant, transcriptome, AabHLH35, abiotic stress

## Abstract

*Anthurium andraeanum* Lind. is a popular potted and cut-flower plant with an attractive spathe and foliage. It is native to tropical rainforest areas and is able to blossom throughout the year under suitable conditions. However, various abiotic stresses seriously restrict the ornamental value of *A. andraeanum* and increase the costs of cultivation. A *dark green* (*dg*) leaf color mutant of *A. andraeanum* ‘Sonate’, which accumulates high levels of anthocyanin, has shown increased vigor and tolerance to stresses during cultivation and is, thus, an ideal germplasm for studying stress tolerance in this species. Here, we show that the anthocyanin content in *dg* mutant plants at different stages of leaf development was higher than in wild-type (WT) plants, and the ability to tolerate under low-temperature (LT, 14 °C) stress was stronger in *dg* than in WT plants. RNA-Seq of cDNA libraries from young leaves of *dg* and WT identified *AabHLH35* as a differentially expressed gene (DEG) that was significantly up-regulated in *dg*. Furthermore, heterologous expression of *AabHLH35* improved tolerance to cold and drought stresses in *Arabidopsis*. These results have built an important molecular foundation for further study of stress tolerance in *A. andraeanum*.

## 1. Introduction

*Anthurium andraeanum* Lind. is a popular potted and cut-flower plant with an attractive spathe and foliage. It is native to tropical rainforest areas and is able to blossom throughout the year under suitable conditions, but abiotic stresses during cultivation, especially cold stress, are serious threats to its growth and development and compromise the economic and ornamental importance of this species. In our previous studies, we acquired a spontaneous leaf color mutant, *dark green* (*dg*), which originated from a leaf color chimera population of tissue culture-derived plantlets in *A. andraeanum* ‘Sonate’ [1]. During natural greenhouse growth, *dg* showed stronger growth vigor and more stress tolerance than wild-type (WT) plants (data not shown). Specifically, the chilling injury traits of WT plants were more obvious than those of *dg* at low temperature environment (Figure S1). However, the molecular mechanism in *dg* that improves plant tolerance to stresses remains elusive.

It has been found that several families of transcription factors (TFs) regulate gene expression in response to stress signals in plants. Among them, three kinds of TFs containing MYB, basic helix-loop-helix (bHLH) and WD repeat (WDR) domains form a complex (designated the MBW complex) that improves plant stress tolerance via an anthocyanin-dependent pathway [2], especially, bHLH proteins belong to one of the largest families of TFs that are involved in tolerance to a variety of abiotic stresses independent of anthocyanin accumulation, including salt [3], cold and freezing [4,5], drought and oxidative stress [5,6]. bHLH proteins contain a highly conserved DNA-binding domain and sequence-specific interaction domain. A typical bHLH domain consists of approximately 60 amino acids that include the basic region and the HLH region [7]. In *Arabidopsis*, *ICE1* (*INDUCER OF CBF EXPRESSION 1*), a *MYC*-like bHLH gene, enhances freezing tolerance through binding to the *CBF3* promoter [4]. *AtbHLH112* is induced by salt, drought and abscisic acid (ABA) and mediates physiological responses to enhance stress tolerance [3]. *AtbHLH68* also has a function in response to drought stress, likely through an ABA-dependent pathway [6]. In addition, bHLHs have been reported to play crucial roles in gene regulation upon abiotic stress in many non-model plants, such as *Citrus sinensis* [5], *Chrysanthemum dichrum* [8], *Phalaenopsis aphrodite* [9], *Brassica campestris* [10], *Fagopyrum tataricum* [11].

In this study, we speculated that some genes, especially TFs, play an important role in the improvement of stress resistance in *dg*. In order to find out the pivotal TFs related to stress tolerance differences between WT and *dg* mutant, we conducted transcriptome analysis through RNA-Seq and identified a differentially expressed TF gene, *AabHLH35*, which had the highest fold-increase in *dg* compared with WT (>17-fold). Further, we found that the *AabHLH35* improved cold and drought stresses tolerance in transgenic *Arabidopsis thaliana*. The present study establishes an important molecular basis for further study of stress tolerance in *A. andraeanum* and is crucial for understanding the leaf color mutation mechanism in foliage plants.

## 2. Results

### 2.1. Effects of Low-Temperature Stress on dg Mutant and WT Plants of A. andraeanum ‘Sonate’

**Figure 1:**
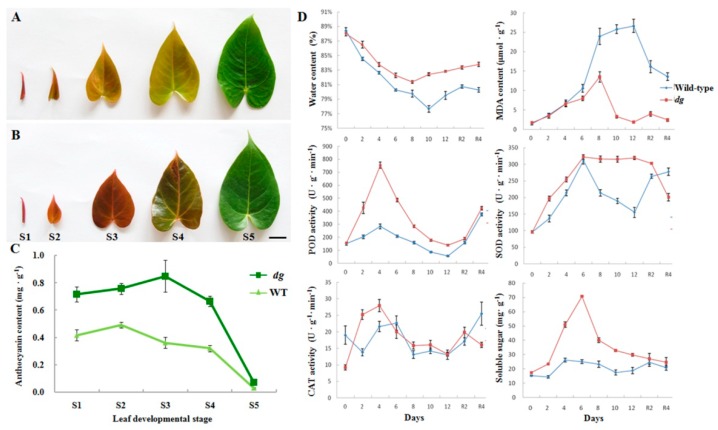
Anthocyanin content in wild-type (WT) and *dg* mutant of *A. andraeanum* ‘Sonate’ and physiological index measurement under low-temperature (14 °C) stress. (**A**,**B**) Leaf developmental stages 1–5 (S1–S5) in WT (**A**) and *dg* mutant (**B**) of *A. andraeanum* ‘Sonate’. Scale bar represents 2 cm. (**C**) Anthocyanin content of WT (light green) and *dg* mutant (*dark green*) at the stages pictured in (**A**,**B**). The error bars in (**C**) represent ± SD (*n* ≥ 3). (**D**) Water content, malondialdehyde (MDA) content, peroxidase (POD) activity, superoxide dismutase (SOD) activity, catalase (CAT) activity and soluble sugar content in leaves of WT (blue lines) and *dg* (red lines) treated at 14 °C for 12 days. Measurements were taken every 2 days (i.e., on day 0, 2, 4, 6, 8, 10 and 12). R2 and R4 indicate plants recovered at 25 °C for 2 and 4 days, respectively. Horizontal coordinates indicate the number of days of low-temperature treatment and recovery. Error bars represent ± SD (*n* = 3).

The leaf anthocyanin content of the *dg* mutant at different developmental stages (stages 1–5) was significantly higher than that in WT, except at stage 5 (Figure 1A–C). Given that anthocyanin production is closely related to stress tolerance, especially cold stress, the effects of low-temperature (LT, 14 °C) stress on *dg* and WT plants of *A. andraeanum* ‘Sonate’ were analyzed. The water content of both *dg* and WT plants decreased until day 8 (*dg*) and day 10 (WT), increased until day 12, and increased further when restored at 25 °C (Figure 1D). The decrease of water content in *dg* (from 88.0% down to 81.4%) was less than that in WT (from 88.5% down to 77.7%), which suggests that *dg* has stronger water retention ability under LT stress (Figure 1D). The malondialdehyde (MDA) level of *dg* at day 12 and after 4 d of restoration at 25 °C was close to the level observed before treatment, whereas the maximum MDA content of WT at 14 °C was twice that of *dg* and did not return to the pre-treatment level even after restoration (Figure 1D). These results showed that the degree of membrane lipid peroxidation in WT was greater than in *dg*, and that LT stress caused more cell membrane damage in WT, while *dg* had a more efficient free radical quenching system and could provide better protection against oxidative stress. The higher levels of peroxidase (POD), superoxide dismutase (SOD), catalase (CAT) and soluble sugars in *dg* relative to WT at most time points indicated that *dg* could adapt to LT stress better than WT through an increase in antioxidant enzyme activity and decrease in membrane damage (Figure 1D).

### 2.2. Analyses of Two Transcriptome Libraries and Differentially Expressed Genes (DEGs) between dg Mutant and WT Plants

In total, 22.2 and 29.8 million clean reads were acquired from the cDNA libraries of WT and *dg* mutant, respectively (Table S1). Among them, 16.1 and 21.1 million mapped reads were obtained from the WT (72.70%) and *dg* (70.73%) libraries, respectively (Table S1). A total of 105,737 transcripts with mean length of 1020.83 nt and 68,179 unigenes with mean length of 777.14 nt were obtained from the two libraries. The N50 lengths of transcripts and unigenes were 1747 and 1352, respectively, indicating their high assembly integrity (Table S2). The majority of unigenes in the two libraries were between 200 and 3000 nt in length, while some were longer than 3000 nt (Figure S2). In total, 24,035 annotated unigenes were ultimately confirmed through alignment of unigene sequences in various databases: the highest frequency of annotated unigenes (98.25%) was found in the Nr database (Figure S3). The Nr database BLAST of *A. andraeanum* unigenes against sequences from known species revealed that 22.52% of the unigenes showed the closest matches with sequences from *Elaeis guineensis*, followed by *Phoenix dactylifera* (20.14%), *Nelumbo nucifera* (10.49%), *Musa acuminate* (8.01%) and *Vitis vinifera* (5.76%) (Figure S4).

In total, we obtained 2565 DEGs, of which 1317 were significantly up-regulated and 1248 were significantly down-regulated in *dg* relative to WT (Figure S5). In a sample of 12 randomly selected DEGs, 5 exhibited lower expression and 7 showed higher expression in *dg* than in WT. All 12 of the selected genes displayed the same expression pattern in the transcriptome data as in qRT-PCR (Figure S6). The Pearson correlation coefficient between the qRT-PCR and RNA-Seq data for the selected genes was 0.866 (*p* < 0.01), suggesting that our transcriptome data were highly credible. Because bHLH-like TFs have important roles in plant stress tolerance, the 7 significantly differentially expressed *bHLH*-like genes from DEGs were examined (Table S3). The three most up-regulated TF genes were annotated as *bHLH35*-like (log_2_FC = 4.15), *HEC1*-like (log_2_FC = 3.55) and *ILR3*-like (log_2_FC = 1.23), and the two down-regulated TF genes were annotated as *bHLH51*-like (log_2_FC = −1.42) and *bHLH113*-like (log_2_FC = −1.40) (Table S3). Two bHLH TFs, ILR3 and MYC2, modulate stress responses under iron deficiency and jasmonate signaling, respectively [12,13]. Moreover, AtbHLH92 functions in plant responses to osmotic stresses in *Arabidopsis* [14]. This suggests that the *bHLH35*-like gene identified here, which was the most up-regulated TF gene in *dg* relative to WT, might have a positive role in stress tolerance.

### 2.3. Isolation of AabHLH35 and Functional Analysis of AabHLH35

**Figure 2:**
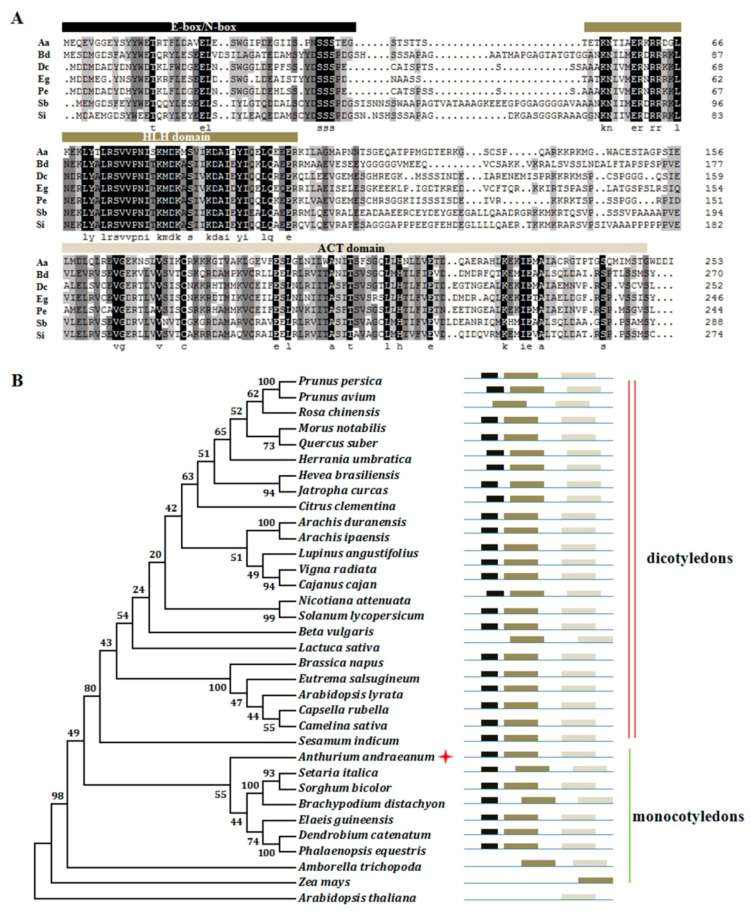
Conserved-domain and phylogenic tree analysis of (helix-loop-helix) bHLH35-like proteins in various species. (**A**) Conserved domains of bHLH35-like proteins. The E-box/N-box, HLH domain and ACT domain are marked by black, green and gray bars, respectively. Aa, *Anthurium andraeanum*; Bd, *Brachypodium distachyon*; Dc, *Dendrobium catenatum*; Eg, *Elaeis guineensis*; Pe, *Phalaenopsis equestris*; Sb, *Sorghum bicolor*; Si, *Setaria italica*. (**B**) Phylogenetic tree of bHLH35-like proteins in various species. Full-length amino acid sequences of bHLH35-like proteins were aligned by ClustalW. The phylogenetic tree was constructed by MEGA 4 according to the Neighbor-Joining method with 1000 bootstrap replicates. Numbers on the tree indicate consensus support values (%). Red star indicates AabHLH35. The colored rectangles correspond to the conserved domains shown in (**A**). The red double line at the right indicates dicotyledons and the green single line indicates monocotyledons.

A coding sequence (CDS) of 765 bp encoding AabHLH35 (c59064.graph_c0) was isolated from *A. andraeanum* leaves. A conserved-domain analysis of the predicted protein sequence showed that AabHLH35 contained the E-box/N-box, HLH domain and ACT domain (Figure 2A). Phylogenetic analysis showed that AabHLH35 was clustered into the same subgroup with other bHLH35-like proteins in monocots, suggesting that AabHLH35 belongs to the bHLH family and that its function in *A. andraeanum* may be similar to that in other species (Figure 2B).

**Figure 3:**
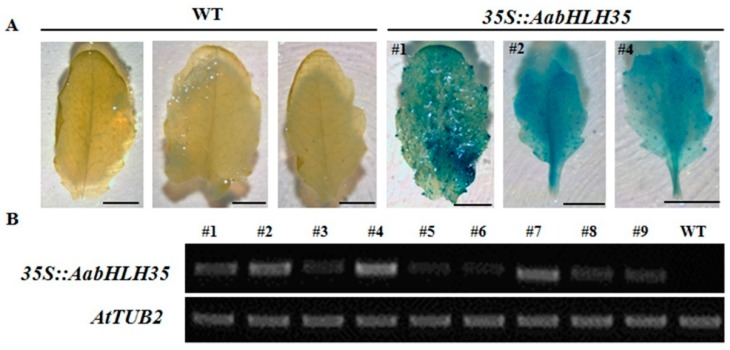
Identification of different *35S::AabHLH35* transgenic lines of *Arabidopsis*. (**A**) Histochemical GUS assays of WT and *35S::AabHLH35* transgenic *Arabidopsis* leaves. (**B**) Expression levels of different *35S::AabHLH35* transgenic lines (#1–#9) determined by semi-quantitative PCR analysis.

To investigate the function of *AabHLH35* under abiotic stress, its coding sequence was heterologous expressed in wild-type *Arabidopsis*. Kanamycin-resistance screening and DNA analysis identified nine independent transgenic lines. GUS assay was performed to further verify the transgenic *Arabidopsis* (Figure 3A). Among them, a *35S::AabHLH35* transgenic line (#4) with strong levels of *AabHLH35* expression was subjected to cold and drought stress treatments (Figure 3B).

**Figure 4:**
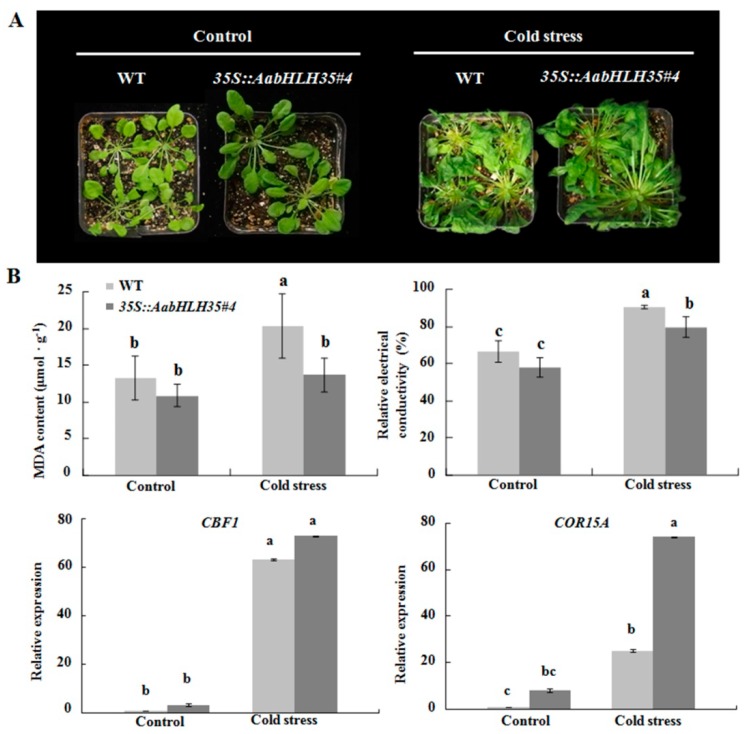
*AabHLH35* improves cold stress in *Arabidopsis*. (**A**) Wild-type (WT) and *35S::AabHLH35 Arabidopsis* plants subjected to cold stress treatment (4 °C for 6 h and 0 °C for 1 h). (**B**) MDA content, relative electrical conductivity and expression levels of *CBF1* and *COR15A* in WT and *35S::AabHLH35 Arabidopsis* plants under cold stress treatment. Error bars represent ± SD (*n* = 3). Letters indicate significant differences (*P* < 0.05, Student’s t-test).

To assess the cold stress tolerance of transgenic *Arabidopsis*, we investigated the phenotypic characteristics of *35S::AabHLH35* and WT plants under cold stress and normal growth condition (22 °C/20 °C, 12 h/12 h). Under normal condition, no significant differences were found between transgenic lines and WT plants (Figure 4A). Under cold stress, the leaves of WT *Arabidopsis* began to turn yellow and showed severe wilting. However, the growth vigor of transgenic plants was significantly better than that of WT plants, although the leaves of *35S::AabHLH35* also displayed wilting to a certain extent (Figure 4A). The MDA content and relative electrical conductivity in cold-stressed *35S::AabHLH35* were significantly lower than those in WT plants, also indicating better cold tolerance (Figure 4B). In *Arabidopsis*, *C-REPEAT/DRE BINDING FACTOR 1* (*CBF1*), a regulator of *COR* (*cold-regulated*) gene, controlled the level of *COR* gene expression, which in turn promoted tolerance to freezing [15]. Heterologous expression of *cold-regulated 15A* (*COR15A*) gene enhanced chilling tolerance in transgenic eggplant (*Solanum melongena* L.) [16]. In this study, the expression of *CBF1* and *COR15A* was significantly up-regulated in both WT and *35S::AabHLH35* transgenic plants under cold stress compared to the unstressed control. Furthermore *COR15A* displayed almost 3-fold higher expression in *35S::AabHLH35* than in WT under cold stress, illustrating that *AabHLH35* may promote *COR15A* expression in response to cold stress (Figure 4B).

**Figure 5:**
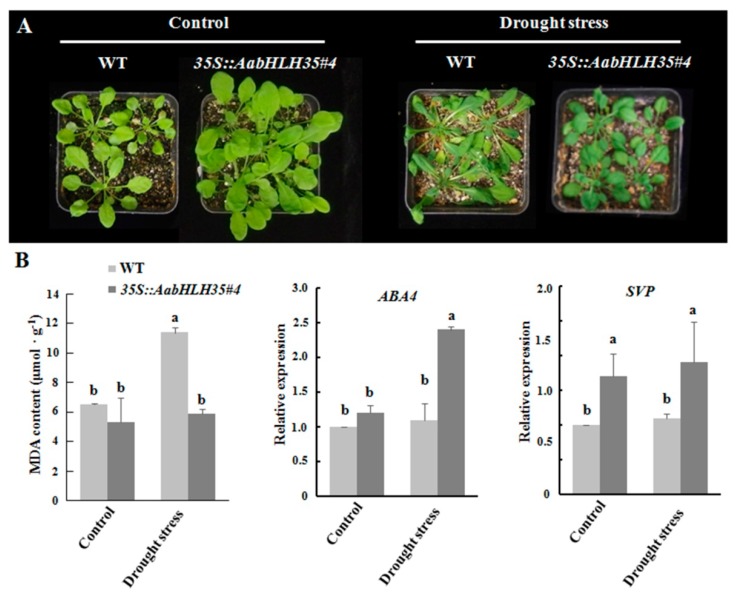
*AabHLH35* improves drought stress in *Arabidopsis*. (**A**) Wild-type (WT) and *35S::AabHLH35 Arabidopsis* plants subjected to drought stress treatment for 15 d. (**B**) MDA content and expression levels of *ABA4* and *SVP* in WT and *35S::AabHLH35 Arabidopsis* plants under drought stress treatment. Error bars represent ± SD (*n* = 3). Letters indicate significant differences (*P* < 0.05, Student’s t-test).

Under drought stress, the leaves of WT displayed more shrinkage, wilting and dehydration than leaves of transgenic line #4 (Figure 5A). The MDA content was significantly lower in *35S::AabHLH35* than in WT under drought stress (Figure 5B). The expression of a drought-responsive gene, *SHORT VEGETATIVE PHASE* (*SVP*) [17], was significantly higher in *35S::AabHLH35* than in WT regardless of drought stress treatment (Figure 5B). Drought stress did not induce any significant change in transcript levels of *ABSCISIC ACID-DEFICIENT 4* (*ABA4*) in WT plants (Figure 5B). However, the expression of *ABA4* was significantly higher in *35S::AabHLH35* under drought stress (Figure 5B). It was previously reported that *aba4* mutants lead to increase water loss rate compared to WT under rapid dehydration, which is consistent with our results on the activation of *ABA4* expression and in turn promotion of tolerance to drought in *35S::AabHLH35* transgenic lines [18]. These results indicated that *AabHLH35* dependent or independent drought stress induced the expression of *ABA4* and *SVP* to improve drought stress tolerance.

## 3. Discussion

In this study, we performed *de novo* transcriptome comparisons between *A. andraeanum dg* mutant and WT plants to identify key DEGs, from which we isolated and characterized a *bHLH35*-like gene, *AabHLH35*. First, we found that anthocyanin contents were much higher in the leaves of *dg* mutant than in WT (Figure 1) and that 11 anthocyanin-metabolism-related genes were significantly up-regulated in *dg* mutant plants relative to WT (Table S4).

Next, we found that the majority of MYB-, bHLH- and WDR-like TFs corresponding to the DEGs (both up- and down-regulated) was involved in plant development and stress response (Table S3). Two bHLH TFs, ILR3 and MYC2, modulate stress responses under iron deficiency and jasmonate signaling, respectively [12,13]. Moreover, *AtbHLH92* functions in plant responses to osmotic stresses in *Arabidopsis* [14]. Notably, *OsbHLH35* was involved in response to salinity stress in rice [19], and overexpressing *PebHLH35* from *Populus euphratica* improved drought tolerance in *Arabidopsis* [20]. In addition, *bHLH35*-like gene expression was positively correlated with anthocyanin accumulation in pear [21]. In the present study, *AabHLH35* displayed 17-fold higher expression in *dg* than in WT (Table S3). Given that both anthocyanin accumulation and cold tolerance in *dg* are stronger than in WT (Figure 1), we speculate that *AabHLH35* plays a major role in stress responses in *A. andraeanum*.

Members of the bHLH TF family were previously reported to regulate anthocyanin accumulation and improve stress tolerance in various species [22,23]. The overexpression of *AabHLH35* in *Arabidopsis* enhanced abiotic stress tolerance, especially, the tolerance to cold and drought stresses in *35S::AabHLH35* transgenic lines was improved compared to that in WT *Arabidopsis* (Figure 4, Figure 5). However, under cold or drought stress, there was no obvious difference in anthocyanin content between transgenic plants and WT (data not shown), illustrating that *AabHLH35* may play a role in stress tolerance through anthocyanin-independent pathways.

## 4. Materials and Methods

### 4.1. Plant Materials and Growth Conditions

The wild-type (normal green type, WT) and leaf color mutant (*dark green* type, *dg* mutant) were grown in a standard greenhouse at Nanjing Agricultural University (Nanjing, Jiangsu Province, China). Spray watering, fertilizer management and disease and pest control were performed using standard methods [24]. Five stages (stages 1–5) of leaves from WT and *dg* were classified on the basis of leaf size and pigment accumulation and used for anthocyanin determination. *A. andraeanum* plants with consistent growth under a 12-h photoperiod (180 μmol m^−2^ s^−1^ light intensity) at 25 °C were used for cold stress treatment (14 °C for 12 days and recovery at 25 °C for 4 days). Samples were frozen in liquid nitrogen and stored at −80 °C until use.

The wild-type *Arabidopsis* Columbia ecotype (Col-0) was used for transgenic plant analysis. Seedlings at the two-true-leaf stage were transplanted into soil and placed in a light incubator under a 12-h photoperiod (150 μmol m^−2^ s^−1^ light intensity) with 22 °C/20 °C light/dark temperatures. As many inflorescences as possible (with the exception of blooming flowers) were used for transformation.

### 4.2. Measurement of Physiological Indexes in A. andraeanum

Anthocyanin extraction was performed as previously described and anthocyanin content was detected by the pH differential method [25,26]. Water content was determined by drying weighing method. The samples were fixed at 105 °C for 30 min and dried at 70 °C until constant weight in the oven. Water content = (W1−W2)/W1 × 100%. W1 stands for leaves fresh weight and W2 for leaves dry weight. MDA content was determined by thiobarbituric acid (TBA) method. 0.1 g of leaves was ground to homogenate by adding 2.0 mL 5% trichloroacetic acid (TCA) and centrifuged at 3000 r min^−1^ for 20 min. And then 1.5 mL supernatant was added 1.5 mL 0.67% TBA solution with mixing well, boiling for 30 min and cooling quickly in ice bath. The absorbance at 600 nm, 532 nm and 450 nm was measured after centrifugation at 3000 r min^−1^ for 10 min at 4 °C. MDA concentration (C) = 6.45 × (OD_532_ − OD_600_) − 0.56 × OD_450_ and MDA content = (C V_T_) / (W V_1_). V_T_ stands for total volume of enzyme solution, W for fresh weight and V_1_ for volume of enzyme solution for determination. POD activity, SOD activity, and CAT activity were determined by guaiacol chromogenic method, photochemical reduction of nitroblue tetrazole (NBT) method and ultraviolet absorption method, respectively. Soluble sugar content was determined by anthrone colorimetry [27]. All measurements were performed on three biological replicates and significance analyses were performed using SPSS 10.0 software (IBM, USA).

### 4.3. RNA Extraction, Library Construction and RNA-Seq Analysis

Total RNA was extracted from WT and *dg* leaves (stage 2) using an improved cetyltrimethylammonium ammonium bromide (CTAB) method [28]. The concentration, purity and integrity of total RNA were identified with a NanoDrop 2000 (Thermo Fisher, USA), Qubit 2.0 (Thermo Fisher, USA) and Agilent 2100 (Agilent, USA), respectively. A total of 1 μg RNA was used for library construction. Sequencing libraries were generated using NEBNext®Ultra™ RNA Library Prep Kit for Illumina®(NEB, USA) following manufacturer’s recommendations and index codes were added to attribute sequences to each sample. Briefly, mRNA was purified from total RNA using poly-T oligo-attached magnetic beads. Fragmentation was carried out using divalent cations under elevated temperature in NEBNext First Strand Synthesis Reaction Buffer (5X). First strand cDNA was synthesized using random hexamer primer and M-MuLV Reverse Transcriptase. Second strand cDNA synthesis was subsequently performed using DNA Polymerase I and RNase H. Remaining overhangs were converted into blunt ends via exonuclease/polymerase activities. After adenylation of 3’ ends of DNA fragments, NEBNext Adaptor with hairpin loop structure were ligated to prepare for hybridization. In order to select cDNA fragments of preferentially 240 bp in length, the library fragments were purified with AMPure XP system (Beckman Coulter, Beverly, USA). Then 3 μl USER Enzyme (NEB, USA) was used with size-selected, adaptor-ligated cDNA at 37 °C for 15 min followed by 5 min at 95 °C before PCR. Then PCR was performed with Phusion High-Fidelity DNA polymerase, Universal PCR primers and Index (X) Primer. At last, PCR products were purified (AMPure XP system) and library quality was assessed on the Agilent Bioanalyzer 2100 system. The libraries were sequenced on an Illumina HiSeq X Ten platform and the paired-end sequencing read length was PE150. The sequencing work was completed by Biomarker Technologies Corporation (Beijing, China).

Raw data (raw reads) of fastq format were firstly processed through in-house perl scripts. In this step, ploy-N and low-quality reads and adapter sequences from the raw data were removed to obtain clean data (clean reads). Meanwhile, Q20, Q30, GC-content and sequence duplication level of the clean data were calculated. All the downstream analyses were based on clean data with high quality. For each sample, the clean reads were assembled using Trinity software with min_kmer_cov set to 2 by default and all other parameters set default to acquire transcript and unigene libraries [29]. The full data sets have been submitted to the Sequence Read Archive (SRA) database of NCBI under accession SRP148842, BioProject: PRJNA472850. The unigenes function was annotated against the NR (NCBI non-redundant protein sequences), Swiss-Prot (A manually annotated and reviewed protein sequence database), GO (Gene Ontology), KOG/COG/eggNOG (Clusters of Orthologous Groups of proteins), KEGG (Kyoto Encyclopedia of Genes and Genomes) and Pfam (Protein family) databases using BLAST [30] with a cutoff of E-value < 1e-05 and HMMER [31] with a cutoff of E-value < 1e−10. Differential expression analysis of two libraries was performed using EBSeq [32]. The significant *p*-value obtained from the original hypothesis test was adjusted by the Benjamini–Hochberg method [33], and finally, the corrected *p*-value, i.e., the FDR (False Discovery Rate), was used as the key index for screening DEGs. FDR < 0.05 & |log2 fold change (FC)| > 1 was set as the threshold for significantly differential expression. The edgeR package was used to draw an MA map of the DEGs [34].

### 4.4. Gene Expression Analysis

SYBR Premix Ex TaqTM II (Til RNaseH Plus) (Takara, Japan) and QuantStudioTM 3 Real-Time PCR Systems (ABI, USA) were used for real-time quantitative reverse transcription PCR (qRT-PCR) analyses. The housekeeping gene *glyceraldehyde-3-phosphate dehydrogenase* (*GAPDH*) was used as an internal reference to normalize the expression data. The 2−^∆∆Ct^ method was used to calculate the relative expression level [35], and the standard deviation was calculated from three biological replicates. The *t*-test (*p* < 0.05) was selected for statistical analysis. Three biological and three technical replicates were performed for each gene. Leaves of *35S::AabHLH35* and wild-type *Arabidopsis* were frozen in liquid nitrogen and stored at −80 °C for semi-quantitative RT-PCR analysis. Total RNA extraction and reverse transcription methods were the same as those used for qRT-PCR. *Arabidopsis tubulin* (*AtTUB2*) and *actin* (*AtActin2*) genes were used as internal controls. Each gene expression analysis was performed at least three times. The gene-specific primers are listed in Supplementary Table S5.

### 4.5. Cloning of AabHLH35 and Amino Acid Sequence Analysis

Total RNA was extracted from young *dg* leaves (stage 2) using a modified CTAB method [28]. cDNA was synthesized using TransScript One-Step gDNA Removal and cDNA Synthesis SuperMix (TransGen, China). The coding sequence (CDS) of *AabHLH35* was obtained using gene-specific primers (Table S5).

The amino acid sequences of related bHLH35-like proteins in various species were obtained by Protein BLAST searches (https://blast.ncbi.nlm.nih.gov/Blast.cgi). The conserved domains analysis was performed with DNAMAN software (Lynnon Biosoft, USA). A phylogenetic tree based on amino acid sequences was constructed using the neighbor-joining (NJ) method via MEGA4 software [36,37].

### 4.6. Heterologous Expression of AabHLH35 and Abiotic Stresses in Arabidopsis

The full-length CDS of *AabHLH35* was amplified by PCR and then inserted into the *Xba*I and *Bam*HI sites of the pBI121 vector, which carries the 35S promoter and β-glucuronidase (GUS) reporter gene. The recombinant plasmid was introduced into *Agrobacterium* strain EHA105 and transformed into *Arabidopsis* wide-type (Col-0) plants through the floral dip method [38]. The transgenic plants were screened on Murashige and Skoog (MS) medium with 75 mg L^−1^ kanamycin and confirmed by GUS assay and PCR amplification assay. The acquired kanamycin-resistant plants were used for subsequent analysis. The primers for vector construction are listed in Table S5.

For cold stress treatment, 4-week-old *35S::AabHLH35*#4 and WT plants grown in soil were placed in a light incubator at 4 °C for 6 h and 0 °C for 1 h. MDA content and relative electrical conductivity (a measure of intracellular electrolyte exosmosis) were detected after resuming growth under a 12-h photoperiod and 22 °C/20 °C light/dark temperatures for 24 h. For drought stress treatment, 3-week-old *35S::AabHLH35*#4 and WT plants grown in soil were placed in a light incubator (22 °C/20 °C light/dark and 12-h photoperiod). One week after growing under normal conditions, the drought treatment was conducted by natural drought (withholding water). Phenotypic observation, MDA content determination and gene expression analysis were performed after withholding water for 15 d.

## 5. Conclusions

The results of this study demonstrated that a bHLH TF, denoted as *AabHLH35*, was identified in *A. andraeanum* through RNA-Seq from WT and *dg* mutant. *AabHLH35* was higher expressed in *dg* than in WT plants, suggesting it may be related to stress resistance as a differential gene. In addition, heterologous expression of CDS (765 bp) of *AabHLH35* in *Arabidopsis* improved cold and drought tolerance, which is useful for improving abiotic stress tolerance in the cultivation and production of *A. andraeanum* and possibly other ornamentals. However, the specific molecular regulation mechanisms in abiotic stress tolerance remain elusive and need further investigation.

## Supplementary Materials

The following are available online at https://www.mdpi.com/2223-7747/8/7/216/s1, Figure S1: The phenotype of wild-type (WT) and *dark green* (*dg*) mutant of *A. andraeanum* ‘Sonate’ under cold stress. Upper row, the WT treated for 0 h (a), 12 h (b), 24 h (c), 48 h (d); Lower row, the *dg* treated for 0 h (e), 12 h (f), 24 h (g), 48 h (h), Figure S2: Unigene length distribution from transcriptome libraries of *A. andraeanum* ‘Sonate’ WT and *dg* mutant, Figure S3: Summary statistics of functional annotation for *A. andraeanum* unigenes in various databases. Statistics include unigenes from both WT and *dg* mutant, Figure S4: Species distribution of Nr database homologs of unigenes from *A. andraeanum* ‘Sonate’, Figure S5: MA map of differentially expressed genes (DEGs) in *dg* vs WT plants. FPKM indicates fragments per kilobase of transcript per million mapped reads, Figure S6: Expression verification of differentially expressed genes (DEGs) in *dg* vs WT by qRT-PCR. The error bars represent ± SD (*n* = 3), Table S1: Summary of transcriptome sequencing data, Table S2: Overview of the assembly results from transcriptome of *A. andraeanum* ‘Sonate’ leaf, Table S3: List of significantly differentially expressed transcription factor genes in WT and *dg* mutant, Table S4: Differentially expressed genes (DEGs) of anthocyanin metabolism pathway between WT and *dg* mutant of *A. andraeanum* ‘Sonate’, Table S5: Primers used in this study.
