# Supplementary material for: The AabHLH35 Transcription Factor Identified from Anthurium andraeanum is Involved in Cold and Drought Tolerance
Source: Plants (Basel). 2019 Jul 11;8(7):216. doi: 10.3390/plants8070216 (PMC6681207; doi:10.3390/plants8070216)
Supplement: Supplementary file 1 [file plants-08-00216-s001.pdf]

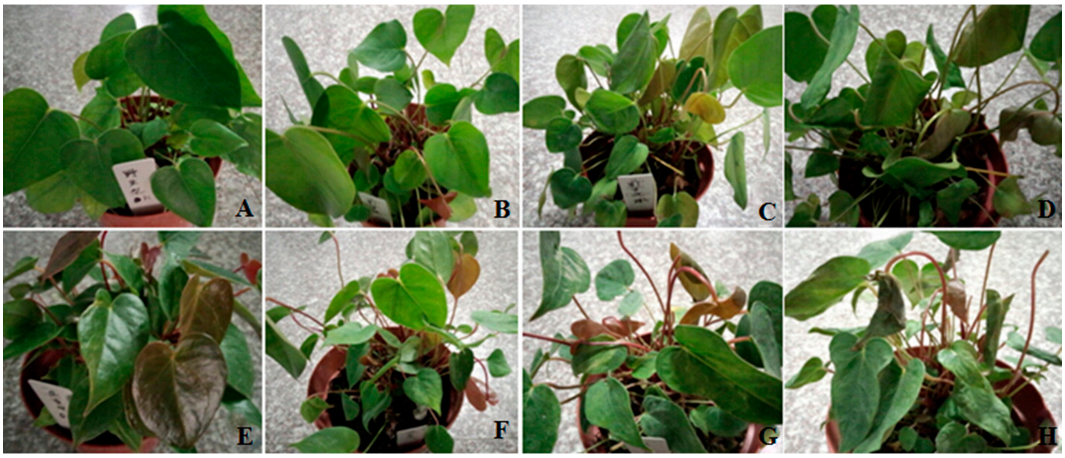

Figure S1. The phenotype of wild-type (WT) and *dark green* (*dg*) mutant of *A. andraeanum* 'Sonate' under cold stress. Upper row, the WT treated for 0 h (a), 12 h (b), 24 h (c), 48 h (d); Lower row, the *dg* treated for 0 h (e), 12 h (f), 24 h (g), 48 h (h).

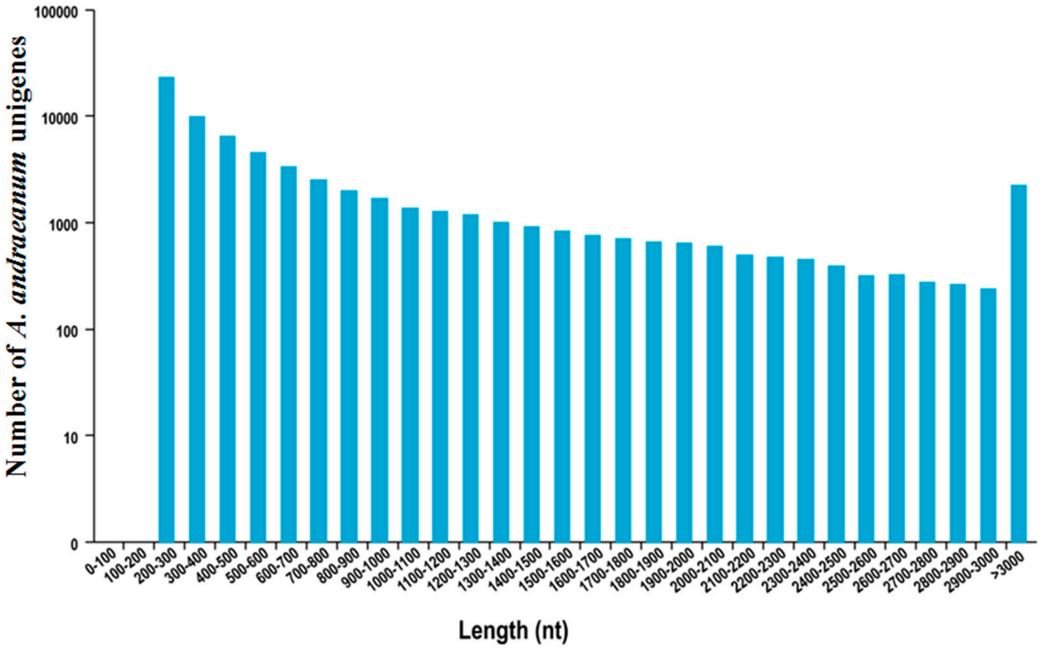

Figure S2. Unigene length distribution from transcriptome libraries of *A. andraeanum* 'Sonate' WT and *dg* mutant.

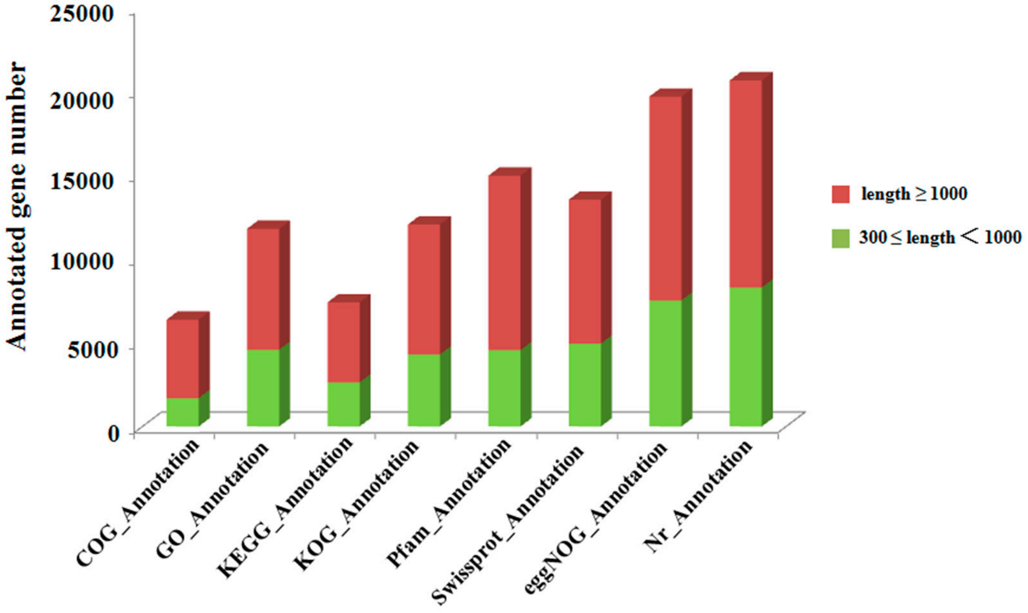

Figure S3. Summary statistics of functional annotation for *A. andraeanum* unigenes in various databases. Statistics include unigenes from both WT and *dg* mutant.

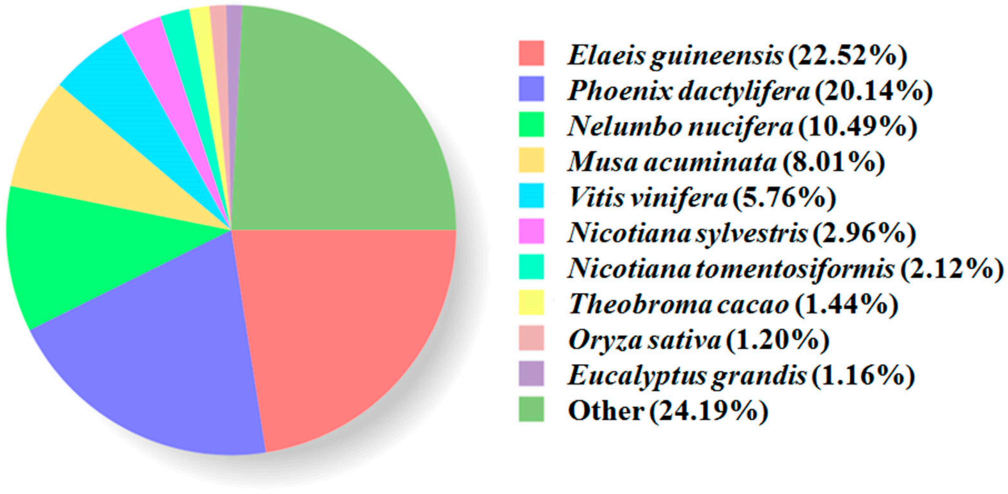

Figure S4. Species distribution of Nr database homologs of unigenes from *A. andraeanum* 'Sonate'.

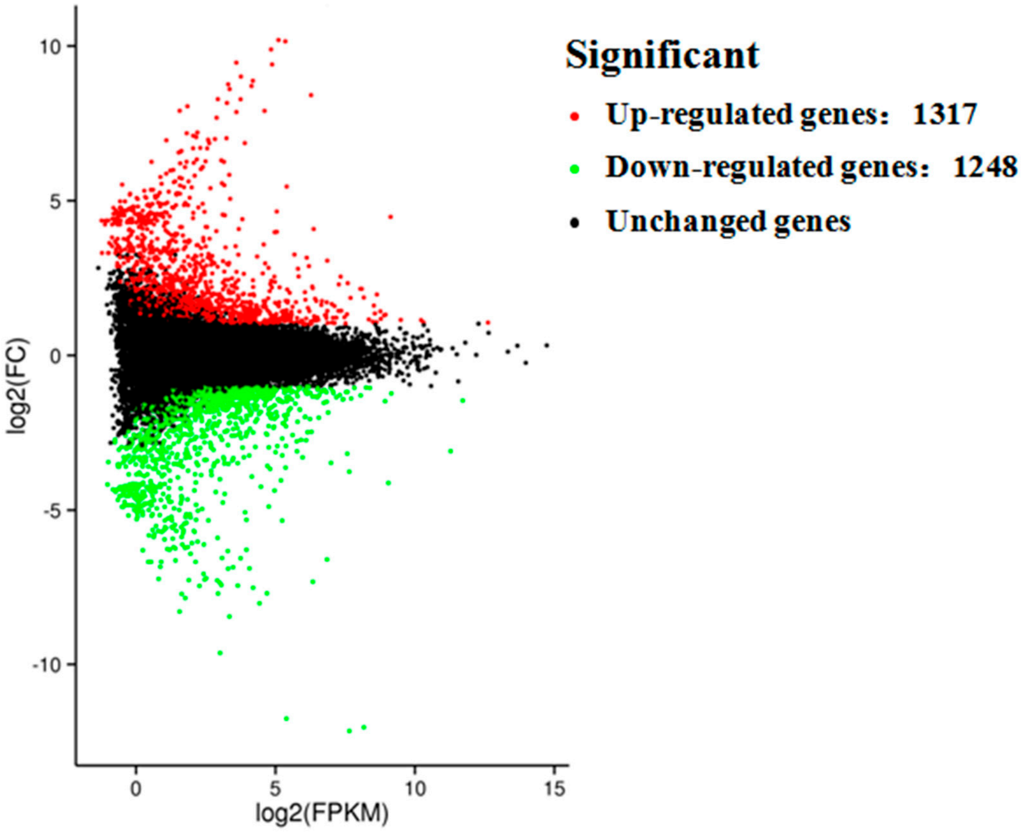

Figure S5. MA map of differentially expressed genes (DEGs) in *dg* vs WT plants. FPKM indicates fragments per kilobase of transcript per million mapped reads.

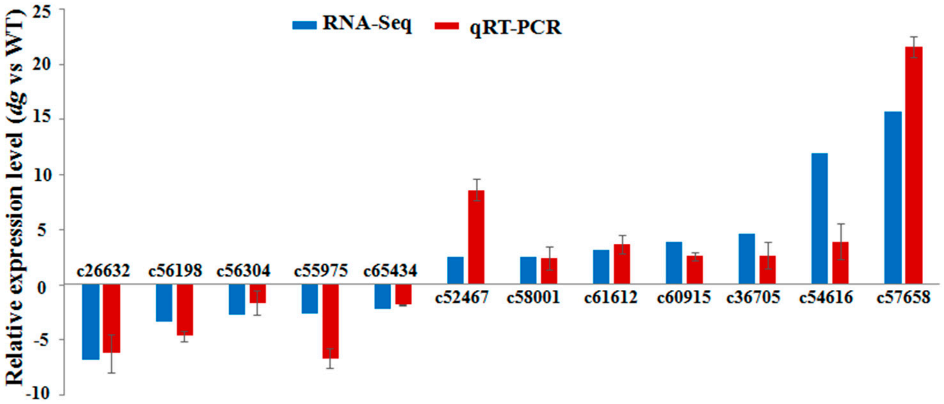

Figure S6. Expression verification of differentially expressed genes (DEGs) in *dg* vs WT by qRT-PCR. The error bars represent ± SD (n = 3).

Table S1. Summary of transcriptome sequencing data.

	Wild-type Leaf	*dark green* Leaf
Clean reads	22,162,297	29,816,817
GC content	54.10%	54.49%
%≥Q30	91.52%	92.02%
Mapped reads	16,112,556	21,089,627
Mapped ratio	72.70%	70.73%

Table S2. Overview of the assembly results from transcriptome of *A. andraeanum* 'Sonate' leaf.

	Contig	Transcript	Unigene
Total number	12,456,845	105,737	68,179
Mean length	44.35	1020.83	777.14
N50 length	45	1,747	1,352

Table S3. List of significantly differentially expressed transcription factor genes in WT and *dg* mutant.

Gene ID	FDR	Log_2 FC (*dg* vs WT)	Putative Function
c59064.graph_c0	0.0	4.15	Transcription factor bHLH35-like
c50234.graph_c0	9.8E−06	3.55	Transcription factor HEC1-like
c60337.graph_c0	4.2E−04	1.23	Transcription factor ILR3-like
c63809.graph_c0	3.0E−04	1.07	Transcription factor bHLH92-like
c61840.graph_c1	4.6E−03	1.01	Transcription factor MYC2-like
c56764.graph_c0	1.4E−03	−1.40	Transcription factor bHLH113-like
c55975.graph_c0	4.5E−03	−1.42	Transcription factor bHLH51-like

FC = fold change.

Table S4. Differentially expressed genes (DEGs) of anthocyanin metabolism pathway between WT and *dg* mutant of *A. andraeanum* 'Sonate'.

Functional Category	Unigene ID	Log_2 FC (*dg* vs WT)	Direction of Regulation	Unigene Annotation
Anthocyanin metabolism	c49466.graph_c0	3.697	Up	3MAT
	c54616.graph_c0	3.584	Up	F3′H
	c66610.graph_c0	2.750	Up	UFGT
	c66417.graph_c0	2.552	Up	F3H
	c25307.graph_c0	1.855	Up	UFGT
	c49585.graph_c0	1.676	Up	UFGT
	c55410.graph_c0	1.589	Up	UFGT
	c59304.graph_c0	1.556	Up	UFGT
	c57371.graph_c0	1.420	Up	F3H
	c58001.graph_c0	1.316	Up	CHS

	c62658.graph_c0	1.266	Up	UFGT
	c56304.graph_c0	−1.473	Down	4CL
	c69569.graph_c0	−5.870	Down	F3′5′H
Anthocyanin-related transport genes	c64798.graph_c2	8.411	Up	GST
	c67645.graph_c1	−1.481	Down	GST
	c51906.graph_c0	−2.727	Down	GST
	c40741.graph_c0	6.583	Up	MATE
	c49595.graph_c0	2.374	Up	MATE
	c34321.graph_c0	1.724	Up	MATE
	c65896.graph_c0	1.706	Up	MATE
	c59554.graph_c0	−2.571	Down	MATE
Other flavonoid metabolism genes	c23443.graph_c0	4.769	Up	HCT
	c60374.graph_c0	1.017	Up	IF7GT

FC = fold change.

Table S5. Primers used in this study.

Gene ID	Primers Name	Sequence
c59064.graph_c0	bHLH35-cF	5′ ATGGAACAGGAAGTGGGAG 3′
	bHLH35-cR	5′ CTAGAAGATGTCATCCCATCCA 3′
	bHLH35-eF	5′ TCTAGA ATGGAACAGGAAGTGGGAG 3′
	bHLH35-eR	5′ GGATCCGAAGATGTCATCCCATCCA 3′
	bHLH35-sqF	5′ GTCGTGCCCAACATCTCC 3′
	bHLH35-sqR	5′ TTGCCATTTCAATCTTCTCCT 3′
NM_125664	AtTUB2-sqF	5′ CCAGGGTGGTCAATGCG 3′
	AtTUB2-sqR	5′ TCCCAGGCTCCAAATCC 3′
AT3g18780	AtActin2-qF	5′ GGTAACATTGTGCTCAGTGGTGG 3′
	AtActin2-qR	5′ AACGACCTTAATCTTCATGCTGC 3′
AT4g25490	AtCBF1-qF	5′ GGCGTTGGCTTTTCAAGATG 3′
	AtCBF1-qR	5′ AAGTCGGCATCCCAAACATT 3′
AT2g42540	AtCOR15A-qF	5′ GCAGATGGTGAGAAAGCGAAAGAC 3′
	AtCOR15A-qR	5′ CGGCTTCTTTTCCTTTCTCCTC 3′
AT2g22540	AtSVP-qF	5′ AACGCTGCTGTGTACGAGGAAG 3′
	AtSVP-qR	5′ TCTCTAACCACCATACGGTAAGCC 3′
AT1g67080	AtABA4-qF	5′ TCTAGTAAATACATGTTGCCAG 3′
	AtABA4-qR	5′ TCTGGTTCTCTAACCCATCATT 3′
JN602203	GAPDH-qF	5′ CCGAGTTCCCACTGTCGATG 3′
	GAPDH-qR	5′ AATGCTCGACCTGCTGTCAC 3′
c26632.graph_c0	CCD-qF	5′ CATCCTAGCTGCCTACCTCCT 3′
	CCD-qR	5′ AAGCGCCATACACGTCCC 3′

c56198.graph_c0	Chl1-qF	5′ CATTGGCTTCGGCTGTTGC 3′
	Chl1-qR	5′ GGAGCTGGCGGTAGTAATTGTTG 3′
c56304.graph_c0	4CL-qF	5′ CGGTGCCCATGTTCCAC 3′
	4CL-qR	5′ CAGATGACCTCCCGCAGC 3′
c55975.graph_c0	bHLH51-qF	5′ TTCCTGCGGGTGGTCTT 3′
	bHLH51-qR	5′ CTCCATCCCCACGGACG 3′
c65434.graph_c0	MYB1-qF	5′ GCTGGGAGGTTGACGAGT 3′
	MYB1-qR	5′ CCATCTGAGCAAGAAGCCAT 3′
c52467.graph_c0	Photo-qF	5′ TCCACCTTCCTGCTCACGC 3′
	Photo-qR	5′ CCCTCTTGGGTCCCTTCG 3′
c58001.graph_c0	CHS-qF	5′ CATCGGCACAGCCAACC 3′
	CHS-qR	5′ CACCTCCACCACCACCAT 3′
c61612.graph_c0	WD40-qF	5′ CCGAGGCCGGGGTCATCTA 3′
	WD40-qR	5′ AGGAGGGGAAGGCGTTGTT 3′
c60915.graph_c0	SPS-qF	5′ ACAATGGCAGATGGACAC 3′
	SPS-qR	5′ GTCTTGTAGGTCAGGGTCA 3′
c36705.graph_c0	SS-qF	5′ CTGGGTTGCTCTGGCTGTT 3′
	SS-qR	5′ GAGGAAAGGATGCGTTGA 3′
c54616.graph_c0	F3′H-qF	5′ GCTGCCTTCCTCAAGACCCAT 3′
	F3′H-qR	5′ AGGTGGACGGAGCAGAGCTT 3′
c57658.graph_c0	Chl2-qF	5′ CACCACCTCAGCCACTCAC 3′
	Chl2-qR	5′ TCTCCTGCACTGTTCTCCAA 3′
